# Use of SNP genotypes to identify carriers of harmful recessive mutations in cattle populations

**DOI:** 10.1186/s12864-016-3218-9

**Published:** 2016-11-03

**Authors:** Filippo Biscarini, Hermann Schwarzenbacher, Hubert Pausch, Ezequiel L. Nicolazzi, Yuri Pirola, Stefano Biffani

**Affiliations:** 1 Department of Bioinformatics and Biostatistics, PTP Science Park, Via Einstein - Loc. Cascina Codazza, Lodi, 26900 Italy; 2 IBBA-CNR, Via Einstein - Loc. Cascina Codazza, Lodi, 26900 Italy; 3 ZuchtData, Dresdner Straße 89/19, Wien, A-1200 Austria; 4 Technische Universität München, Liesel-Beckmann-Straße 1, Freising-Weihenstephan, D-85354 Germany; 5 DISCo, Università degli Studi di Milano-Bicocca, Viale Sarca 336, Milano, Italy

**Keywords:** SNP genotypes, Recessive mutations, Carrier identification, Lasso-penalised logistic regression, support vector machines, KNN, MAG, Haplotypes, Cattle

## Abstract

**Background:**

SNP (single nucleotide polymorphisms) genotype data are increasingly available in cattle populations and, among other things, can be used to predict carriers of specific mutations. It is therefore convenient to have a practical statistical method for the accurate classification of individuals into carriers and non-carriers. In this paper, we compared – through cross-validation– five classification models (Lasso-penalized logistic regression –Lasso, Support Vector Machines with either linear or radial kernel –SVML and SVMR, k-nearest neighbors –KNN, and multi-allelic gene prediction –MAG), for the identification of carriers of the *TUBD1* recessive mutation on BTA19 (Bos taurus autosome 19), known to be associated with high calf mortality. A population of 3116 Fleckvieh and 392 Brown Swiss animals genotyped with the 54K SNP-chip was available for the analysis.

**Results:**

In general, the use of SNP genotypes proved to be very effective for the identification of mutation carriers. The best predictive models were Lasso, SVML and MAG, with an average error rate, respectively, of 0.2 *%*, 0.4 *%* and 0.6 *%* in Fleckvieh, and 1.2 *%*, 0.9 *%* and 1.7 *%* in Brown Swiss. For the three models, the false positive rate was, respectively, 0.1 *%*, 0.1 *%* and 0.2 *%* in Fleckvieh, and 3.0 *%*, 2.4 *%* and 1.6 *%* in Brown Swiss; the false negative rate was 4.4 *%*, 7.6 *%*1.0 *%* in Fleckvieh, and 0.0 *%*, 0.1*%* and 0.8 *%* in Brown Swiss. MAG appeared to be more robust to sample size reduction: with 25 *%* of the data, the average error rate was 0.7 *%* and 2.2 *%* in Fleckvieh and Brown Swiss, compared to 2.1 *%* and 5.5 *%* with Lasso, and 2.6 *%* and 12.0 *%* with SVML.

**Conclusions:**

The use of SNP genotypes is a very effective and efficient technique for the identification of mutation carriers in cattle populations. Very few misclassifications were observed, overall and both in the carriers and non-carriers classes. This indicates that this is a very reliable approach for potential applications in cattle breeding.

## Background

Several monogenic (Mendelian) mutations have been revealed in the cattle genome (e.g. [1, 2]): although some are useful (e.g. casein variants [3]), most of such mutations are harmful (e.g. [4, 5] for a review). Dominant mutations can easily be purged from the population, since carriers are easily identified and excluded from the breeding stock. Recessive mutations, on the other hand, are more difficult to manage: a small proportion of carriers is bound to remain in the population. In case of phenotypic selection (natural or artificial) against homozygotes (animals showing the defect), the frequency of such carriers would asymptotycally approach zero, and the occurrence of the disorder would be a rare event. When top-ranked bulls for relevant breeding objectives are carriers, though, higher frequencies of the mutation remain in the population and the related genetic disorder becomes a more serious issue. This is critical in domesticated cattle -especially in highly specialized dairy breeds- given their specific population structure: high inbreeding, small number of founder animals, declining effective population size and widespread use of artificial insemination, all contribute to making modern cattle breeds particularly susceptible to recessive genetic disorders [6]. Examples of harmful recessive mutations in cattle include some long known mutations like BLAD (Bovine Leukocyte Adhesion Deficiency) [7] and CVM (Complex Vertebral Malformation) [8], and some recently detected mutations like the one in the *CWC15* gene on BTA15 (haplotype JH1) in Jersey cattle [9].

In the case of harmful recessive mutations, it is essential to identify carriers in order to remove them from the breeding population, or to apply effective mating strategies to counteract the diffusion of the undesired allele and keep its frequency low. The causal mutation of a harmful defect may be already known (as is the case of *CWC15*, or of the *Weaver syndrome* [10]) or not yet (for example the mutation behind syndactyly in Holsteins [11]): in this latter case, haplotypes associated with the defect can be detected [12, 13] (e.g. the HHM haplotype associated to syndactyly). Such haplotypes may be more or less tightly associated with the underlying mutation: sometimes the association is almost indissoluble as is between the JH1 haplotype and the CWC15 mutation in Jerseys (99.3 *%*). Some other times it is less reliable, as for instance between the HHC haplotype on BTA3 and CVM: two versions of the haplotype exist, one with and one without the causative mutation [9].

The identification of specific mutations or haplotypes carried by animals is traditionally performed through specific laboratory assays in individual animals. Examples include: microsatellite markers [14] or, in cows, milk isoforms for casein variants [15]; specific gene tests for CVM [16]. These methods, though accurate, can be expensive and time-consuming. Haplotypes can be reconstructed in silico from marker genotypes and pedigree records [17, 18], and have been used successfully for the prediction of *κ*-casein alleles [19]. Pedigree records, though, are not always available, and add to the complexity of the analysis. Whole-genome SNP data are a most valuable source of information and can offer a low-cost and convenient alternative. SNP genotypes (e.g. from SNP chips or genotyping-by-sequencing –GBS– experiments) are increasingly available for large numbers of animals, as a consequence of genomic selection programmes and research projects, and can be used effectively to predict haplotypes or gene alleles of interest. SNP genotypes can be used as they are, or haplotypes can be reconstructed without pedigree information (e.g. they are often readily available from imputation software like “Beagle” [20]). In a previous study the successful identification of carriers of the BH2 haplotype from SNP genotypes only was described [21].

In this work, the use of SNP genotypes alone for the identification of carriers of harmful recessive mutations in cattle is presented. Five classification methods were compared in two cattle breeds with different population and genetic structure, and with opposite carriers to non-carriers ratio. The causative mutation behind the BH2 haplotype [12, 22] was used for illustration. This has been recently demonstrated to be a missense mutation in the gene *TUBD1*, and is linked to high juvenile mortality in the Brown Swiss and Fleckvieh cattle breeds [23].

## Methods

### Animal population, carrier status and SNP genotypes

A population of 3508 bulls and cows from two cattle breeds was used for this study: 3116 Fleckvieh (3103 males, 13 females) and 392 Brown Swiss (333 males, 59 females) animals from farms in Austria and Southern Germany (Bayern). Fleckvieh is a dual-purpose breed (milk and meat production), Brown Swiss is a specialised dairy breed.

All animals were genotyped with the BovineSNP50 v2 (54 K) Illumina BeadChip. Only the 1512 SNPs on BTA19 (*Bos taurus* autosome 19) were used for the analysis. The missing-rate was 5.78 *%* in the Fleckvieh and 4.92 *%* in the Brown Swiss. No individual animal had a call-rate lower than 95 *%*; SNPs with a call-rate <95 *%* were removed from the analysis (195 and 142 SNPs in Fleckvieh and Brown Swiss respectively). Residual missing genotypes were imputed based on linkage disequilibrium, using the localized haplotype clustering imputation method implemented in the computer package “Beagle” v.3 ([20]). After imputation, average MAF (minor allele frequency) was 0.224 and 0.187 in the Fleckvieh and Brown Swiss population respectively.

A direct gene test was performed on all animals to determine carrier status for the *TUBD1* mutation. Genotypes at the mutation site were obtained using a KASP genotyping assay carried out at the laboratory of the Technische Universität München (Freising, Germany: see [23] for details). The mutation of interest was a *T > C* substitution in the coding region of the *TUBD1* gene, at SNP rs383232842, located at the beginning of BTA19 (at 11 063 520 bps on the UMD 3.1 bovine genome assembly). This is the mutation underlying the BH2 haplotype in Brown Swiss and Fleckvieh cattle [13], and has been reported to be associated with stillbirth and low calf survival rate (e.g. [22]). The degree of association between the BH2 haplotype and the mutation *TUBD1* is 99.2 % [23]. The mutation causes the substitution of a histidin by an arginine in the *Tubulin delta 1* protein. The function of the protein is damaged, which is thought to lead to defective cilia in the respiratory tract and, consequently, to chronic airway disease in calves. Animals were identified as carriers (coded as 1) or not (coded as 0) of the mutation. There were 126 (4.04 *%*) and 250 (63.78 *%*) carriers in the Fleckvieh and Brown Swiss datasets, respectively. Data were therefore unbalanced in different directions in the two breeds (more carriers than non-carriers in Brown Swiss, the other way around in Fleckvieh). Table 1 summarizes the animal population and SNP data.

**Table 1:** Description of cattle populations, SNP marker genotypes and carrier status with respect to the *TUBD1* mutation on BTA19

	Fleckvieh	Brown Swiss
N. animals	3116	392
Mutation carriers	126	250
% carriers	4.04 *%*	63.78 *%*
% non-carriers	95.96 *%*	36.22 *%*
SNPs on BTA19	1512	1512
missing rate	5.78 *%*	4.92 *%*
SNPs with call-rate <95 *%*	195	142
SNPs used	1317	1370
average MAF	22.4 *%*	18.7 *%*

All animals were genotyped with the Illumina 54 K SNP-chip v2; only SNPs on BTA19 were used in the analysis

### Identification of mutation carriers

**Fig. 1:**
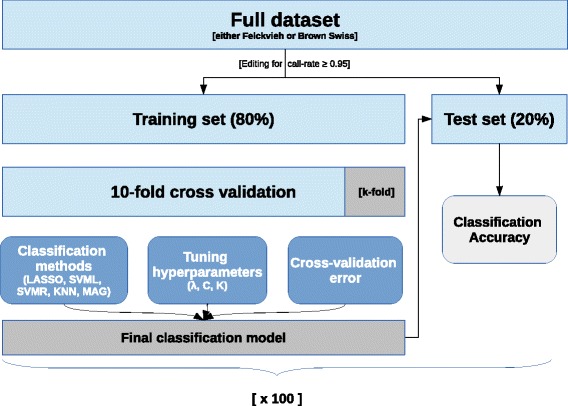
Procedure for the identification of mutation carriers. First data were randomly split into training and test set. Five classifiers (LASSO, SVML, SVMR, KNN, MAG) were trained through a 10-fold cross-validation. The final classification models were applied to the test set for the estimation of the classification accuracy. This process was repeated 100 times

The identification of mutation carriers from SNP genotypes was carried out separately in the two breeds. Two parallel sets of analysis were therefore conducted. First, data were randomly split into a test set and a training set. The test set was kept aside, and used only in the end to test the accuracy of the predictive model. The training set was used to build the predictive model: a 10-fold cross validation scheme was adopted to tune hyperparameters, select variables and estimate parameter coefficients. Five classification methods were tested: four methods that just use single SNP loci as they are: Lasso-penalized logistic regression, support vector machines (SVM) with linear or radial kernel, K-nearest neighbor (KNN); and one method that builds on haplotype reconstructed from SNP genotypes: multi-allelic gene prediction (MAG: [24]). Figure. 1 summarizes the procedure.

#### Classification methods

**Lasso-penalized logistic regression:** The Lasso (least absolute shrinkage and selection operator) is a regularization method for regression models [25]. The Lasso imposes an *ℓ*_1_-norm constraint on the vector of model coefficients, thereby shrinking some parameter estimates to zero and thus selecting variables in or out of the model. The starting point is a logistic regression model for the probability of carrying the mutation in a generalized linear model framework with logit link function:

1 $$ {}logit(p(x_{i}))=log \left(\frac{p(x_{i})}{1-p(x_{i})}\right)=\mu+\sum\limits_{j=1}^{m}z_{ij}SNP_{j}  $$

where *p*(*x*_*i*_) is the *P*(*Y*=1|*X*) for individual *i* with vector of predictors *x*_*i*_; *SNP*_*j*_ is the effect of the *j*_*th*_ SNP marker; *z*_*ij*_ is the genotype of individual *i* at locus *j* (0, 1 or 2 for AA, AB, and BB genotypes). The model in Eq. 1 was fitted by maximizing the corresponding Lasso-penalized log likelihood function [26]:

2 $$ {{\begin{aligned} {}\mathcal{L}(SNP|y,X)=\sum\limits_{i=1}^{n}\left[y_{i}log(p_{i})+(1-y_{i})log(1-p_{i})\right]-\lambda\sum\limits_{j=1}^{m}|SNP_{j}| \end{aligned}}}  $$

The rightmost term in Eq. 2 is the Lasso (*ℓ*_1_) penalization; the tuning parameter *λ* controls the degree of regularization, and was specified through cross-validation. Logistic regression returns the log-odds of *p*(*x*) which are backtransformed to *P*(*Y*=1|*X*) through the cumulative distribution function of the logistic distribution (i.e. the logistic function). Individuals with *p*(*x*)>/<0.5 were classified as carriers or not of the mutation.

The Lasso, by setting some of the coefficients to be equal to zero, performs variable selection, thus yielding sparse models involving only a subset of the original variables. Lasso regularization is therefore particularly appropriate when many of the predictor coefficients are indeed expected to truly be close or equal to zero. This may apply well to the modelling of mutation carrier status as function of SNP genotypes, since SNP close to the mutation are expected to contribute largely to the prediction accuracy [27]. A regularization method like the Lasso estimator was needed for Brown Swiss, where the number of SNPs exceeds the number of animals (*p*>*n*). In the Fleckvieh, though not required, still the Lasso estimator may be helpful for the interpretation of the model.

**SVM:** Two support vector machines (SVM) models were fitted for the classification of carriers and non-carriers of the mutation: with linear (SVML, see Eqs. 3 and (6)) and radial (SVMR, see Eqs. 3 and (7)) kernel functions. In binary classification problems, SVM attempts at separating classes by a *p*−1 dimensional hyperplane. SVM maps the vector of SNP genotypes $\mathbf {x} \in \mathbb {R}$ into a higher dimensional feature space $\phi (\mathbf {x}) \in \mathbb {H}$ and constructs a separating hyperplane -linear in $\mathbb {R}$- which maximises the margin *M*, the distance between the hyperplane and the nearest data points. The mapping is performed by a kernel function *K*(*x*_*i*_,*x*_*j*_)=〈*h*(*x*_*i*_),*h*(*x*_*j*_)〉 which defines an inner product of some functions *h*(·) of pairs of observations in the space $\mathbb {H}$ (a full description of SVM can be found in [28, 29]). A kernel is a function that quantifies the similarity between observations. In its basic form, the SVM classifier applies the kernel function *K* to all $N \choose 2$ pairs of observations, and returns the following linear (in $\mathbb {R}$) classifier for any observed feature vector *x*:

3 $$ f(x)=\beta_{0}+\sum\limits_{i \in N}\alpha_{i} K(x,x_{i})  $$

The intercept *β*_0_ and coefficients *α*_*i*_ are obtained by maximizing the margin around the separating hyperplane subject to i) allowing some observations to violate the margin *M* (or even fall on the incorrect side of the hyperplane) by a ii) positive quantity *ξ*, and iii) constraining such violations and misclassifications to sum up to a constant *C*:

4 $$ {{\begin{aligned} {}\max_{\beta_{0},\alpha,\xi} M ~\text{subject to} ~\left\{\!\! \begin{array}{rl} \text{i)} & y_{i}\left(\beta_{0}+\sum_{i=1}^{N}\alpha_{i}K(x,x_{i})\right) \!\geq\! M(1-\xi_{i}) \quad \!\forall i \\ \text{ii)} & \xi_{i} \geq 0\\ \text{iii)} & \sum \xi_{i} \leq C \end{array} \right. \end{aligned}}}  $$

This is a convex optimization problem that is solved with the method of Lagrange multipliers [30] by re-expressing all parameters in terms of the *α* multipliers in the Lagrange dual function:

5 $$ L_{D}=\sum\limits_{i=1}^{N} \alpha_{i} - \frac{1}{2} \sum\limits_{i=1}^{N} \sum\limits_{j=1}^{N} \alpha_{i}\alpha_{j}y_{i}y_{j}\langle(h(x_{i}),h(x_{j})\rangle  $$

The maximization of *L*_*D*_ yields the solutions for the coefficients **α**, which in turn give us the SVM classifier in Eq. 3. An interesting properties of the SVM classifier is that *α*_*i*_≠0 only for observations that lie on or within the margin *M*, the support vectors, thus making SVM computations relatively inexpensive.

When *h*(*x*)=*x*, we have the following linear kernel:

6 $$ K(x_{i},x_{i'})=\langle h(x_{i}),h(x_{i'}) \rangle = \langle x_{i},x_{i'} \rangle = \sum\limits_{j=1}^{p} x_{ij} \cdot x_{i'j}  $$

where *p* are the parameters (SNP) in the model. The linear kernel quantifies the similarity between observations using Pearson correlations and the corresponding SVM classifier is equivalent to the support vector classifier [31]. The linear kernel produces a linear decision boundary between classes. Non-linear decision boundaries can be obtained using more complex kernel functions, like in the radial kernel:

7 $$ K(x_{i},x_{i'})=\exp\left(-\gamma \sum\limits_{j=1}^{p}(x_{ij}-x_{i'j})^{2}\right)  $$

where the *x*_*ij*_’s are again 0/1/2-coded SNP genotypes. For both SVML and SVMR the constant *C* defines how much the margin *M* can be violated when building the SVM classifier; *C* is therefore a tuning parameter that controls the bias/variance trade-off in SVM classification.

**KNN:** k-nearest neighbors (KNN) is a general non-parametric classification method. For any given observation *x*_0_, Euclidean distances based on SNP genotypes were calculated to identify the *K* nearest observations defining the neighborhood $\mathcal {N}_{0}$ (the “neighbors”). The conditional probability of carrying or not the mutation was then estimated as the fraction of carriers/non-carriers in $\mathcal {N}_{0}$:

8 $$ P(Y=[0/1]|X=x_{0})=\frac{1}{K}\sum\limits_{i \in \mathcal{N}_{0}}I\left(y_{i}=[0/1]\right)  $$

The observation *x*_0_ was then assigned to the class (carrier/non-carrier) with the largest probability from Eq. 8.

**MAG:** Multi-allelic gene prediction (MAG, [24]) is a method specifically aimed at predicting gene alleles from SNP data. As a consequence, it is significantly different from the previously illustrated methods, that are general classification algorithms, and is a representative of the general class of haplotype-based prediction methods. MAG was developed for the prediction of HLA alleles in humans, but has been previously applied to the prediction of k-casein alleles in cattle [19].

Given the SNP genotype *g*_*i*_=(*gi*1′*gi*1″,…,*gim*′*gim*″) and the gene genotype *h*_*i*_=*hi*′*hi*″ of each individual *i* of a training population *T*, the method estimates the frequencies Pr(*hi*′*Gi*′) and Pr(*hi*″*Gi*″) of the *extended haplotypes* $h^{\prime }_{i}G'_{i}$ and *hi*″*Gi*″, where $G^{\prime }_{i}$ and *Gi*″ are pairs of SNP haplotypes consistent with the observed SNP genotype *g*_*i*_. This estimation is performed, assuming Hardy-Weinberg equilibrium, by maximizing the following log likelihood function:

$$\sum\limits_{i \in T} \log \left(\sum\limits_{(h'_{i}G'_{i}, h^{\prime\prime}_{i}G^{\prime\prime}_{i}) \in \Omega(h_{i}, g_{i})} \Pr(h'_{i}G'_{i}) \cdot \Pr(h^{\prime\prime}_{i}G^{\prime\prime}_{i}) \right) $$

where *Ω*(*h*_*i*_,*g*_*i*_) denotes the set of extended haplotypes pairs consistent with the observed genotypes. Standard maximization methods (such as Expectation-Maximization) are computationally demanding since the number of haplotypes consistent with the observed genotypes increases at exponential rate as the number of SNPs increases. MAG, instead, is based on an estimating equation approach developed by the same authors that makes computationally more efficient the covariance matrix computation component without invalidating the consistency of the computed haplotype probabilities.

Once the extended haplotype probabilities have been estimated, the prediction is simply performed by computing the gene genotype *h* ^′^ *h* ^″^ that maximizes the probability of having such a gene genotype given the observed SNP genotypes using the Bayes’ theorem.

For the present application of MAG to predicting mutation carriers, carrier and non-carrier status were encoded as heterozygous 01 and homozygous 00 gene genotypes, respectively.

#### Tuning hyperparameters and measuring prediction accuracy

The data were partitioned into training and test datasets: 80 *%* of the observations (∼2492 Fleckvieh, ∼313 Brown Swiss) were used to train the predictive model; the remaining 20 *%* of the data (∼624 Fleckvieh, ∼79 Brown Swiss) was set aside and used only to measure the classification accuracy. A 10-fold cross-validation scheme was applied to the training data in order to tune the hyperparameters (*λ*, *C*, *K*) and estimate the effects of the model. The training data were split in 10 subsets of approximately equal size. The first subset was treated as validation set, while the model was fit on the remaining 9 subsets (the training set). Prior to fitting the model, monomorphic and collinear SNPs were edited out of the training set, to get rid of non-informative and redundant predictors and avoid problems due to linear dependecies. In turn, each of the 10 subsets was used as validation set, so that in the end every observation was used both to train and validate the model. The final classification model was then applied to the test set to estimate the accuracy of identifying carriers of the mutation in independent data. These procedure was repeated 100 times (10-fold CV x 100), each time resampling different training and test sets, eventually yielding 100 replicates of the analysis (per breed, per model). The procedure is illustrated in Fig. 1.

The error rate was estimated as the fraction of misclassified observations: $ER=\frac {1}{n} \sum _{i=1}^{n} I(y_{i} \neq \hat {y_{i}})$. Three different error rates were measured: 1) the Total Error Rate (TER) defined as the total number of misclassifications over the total test sample size; 2) the False Positive Rate (FPR) defined as the number of non-carriers misclassified as carriers over the total number of non carriers; and 3) the False Negative Rate (FNR) defined as the number of carriers misclassified as non-carriers over the total number of carriers. TER, FPR and FNR were averaged over the 100 replicates to estimate the test error and the variability of the prediction accuracy.

### Software and computation resources

Data preparation and editing, and all statistical analysis were performed using the *R* programming environment v.3.2.3 [32], except missing genotype imputation, which was carried out with the computer package “Beagle” v.3.3.2 [20]. The R packages *glmnet* [33], *e1071* [34] and *class* were used to fit the Lasso logistic regression, SVM with linear and radial kernels and KNN classification models. MAG has been performed using the MATLAB-based program provided by the authors on their website (http://www.mybiosoftware.com/magprediction-gene-allele-prediction-unphased-snp-data.html). The analyses were run on the bioinformatics platform at PTP Science Park (www.ptp.it), which includes a high performance computing cluster with 600 CPUs, 2.5 TB of RAM and 100 TB of storage space for archiving and back-up.

## Results

The accuracy of identifying carriers of the *TUBD1* mutation on BTA19 was measured over all animals and per class (carriers and non-carriers) for each of the five classification methods, in Brown Swiss and Fleckvieh separately. Average values over 100 replicates are presented. The total predictive accuracy (TPA=1-TER) ranged between 0.837 and 0.991 in Brown Swiss, and between 0.970 and 0.998 in Fleckvieh. The accuracy among carriers (true positive rate: TPR=1-FNR) was in the range 0.862 - 1.000 in Brown Swiss and 0.288 - 0.991 in Fleckvieh. The accuracy among non-carriers (true negative rate: TNR=1-FPR) varied between 0.798 and 0.984 in Brown Swiss, and between 0.998 and 0.999 in Fleckvieh.

Lasso penalized logistic regression, SVML and MAG consistently gave the highest accuracy in both classes and breeds, while KNN was always the worst performing classification method. MAG showed the highest accuracy in the minority class in both breeds (highest TNR in Brown Swiss, highest TPR in Fleckvieh). Table 2 summarizes the predictive accuracy over methods and breeds.

**Table 2:** Total prediction accuracy (TPA), true positive rate (TPR) and true negative rate (TNR) with kNN, Lasso, SVML and SVMR in Brown Swiss and Fleckvieh cattle

	Method	TPA	TPR	TNR
Brown	KNN	0.837 (±0.047)	0.862 (±0.062)	0.798 (±0.088)
	Lasso	0.988 (±0.010)	1.000 (±0.000)	0.968 (±0.029)
	SVML	0.991 (±0.010)	0.998 (±0.005)	0.976 (±0.025)
	SVMR	0.978 (±0.016)	0.989 (±0.015)	0.961 (±0.034)
	MAG	0.983 (±0.015)	0.992 (±0.011)	0.984 (±0.016)
Fleckvieh	KNN	0.970 (±0.007)	0.288 (±0.087)	0.998 (±0.002)
	Lasso	0.998 (±0.002)	0.957 (±0.037)	0.999 (±0.001)
	SVML	0.996 (±0.002)	0.924 (±0.047)	0.999 (±0.001)
	SVMR	0.991 (±0.003)	0.775 (±0.073)	0.999 (±0.001)
	MAG	0.994 (±0.003)	0.991 (±0.003)	0.998 (±0.002)

*KNN* k-nearest neighbors, *Lasso* *ℓ*1-penalized logistic regression, *SVML* suport vector machine with linear kernel, *SVMR* support vector machine with radial kernel *MAG* multi-allelic gene prediction

**Fig. 2:**
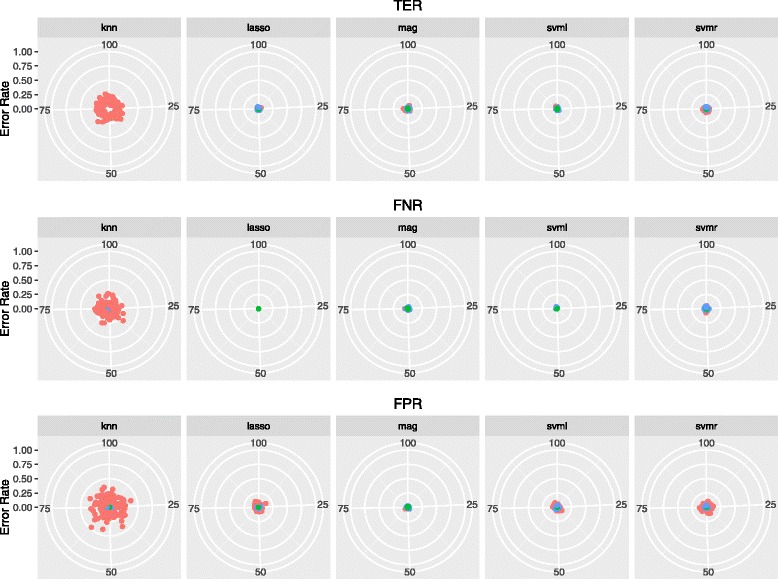
Accuracy of identifying mutation carriers in Brown Swiss cattle. TPA: total prediction accuracy; TNR: true negative rate; TPR: true positive rate. Reported accuracy are obtained from 10-fold cross-validation repeated 100 times

**Fig. 3:**
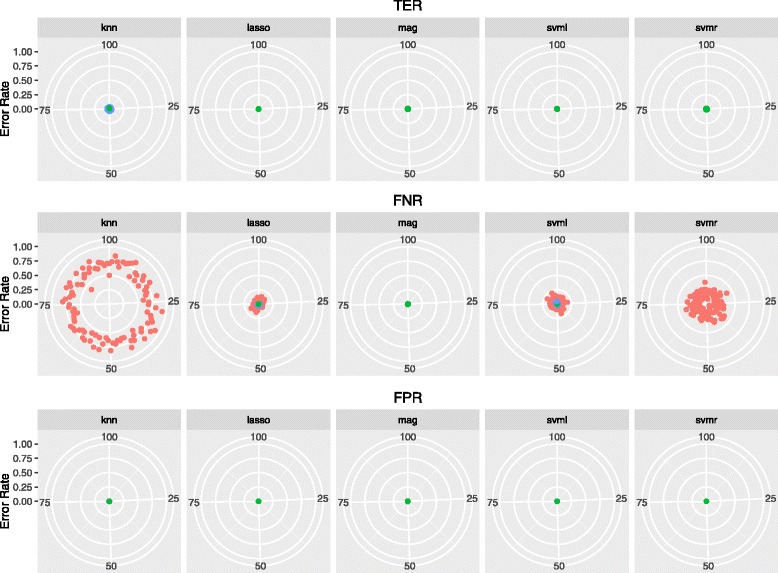
Accuracy of identifying mutation carriers in Fleckvieh cattle. TER: total error rate; FPR: false positive rate; FNR: false negative rate rate. Reported accuracy are obtained from 10-fold cross-validation repeated 100 times

Figures 2 and 3 show the error rates (TER, FPR, FNR) for each of the 100 replicates of the analysis in Brown Swiss and Fleckvieh, thereby visualizing the variability of prediction: the closer to the center of the target, the lower the prediction error. The error rate was generally low, with very limited variability. The error was lower than 5 *%* (with standard deviation ≤4 percentage points – *pp*) in all cases except all KNN predictions in Brown Swiss (TER, FNR and FPR: 16.3 *%*±4.8*pp*, 13.8 *%*±8.6*pp* and 20.6 *%*±6.2*pp*), and FNR with KNN (71.2 *%*±8.7*pp*), SVML (7.58 *%*±4.7 *pp*) and SVMR (22.5 *%*±7.3 *pp*) in Fleckvieh.

As for hyperparameters, the average *λ* (Lasso penalty), *C*_*SVML*_, *C*_*SVMR*_ (SVM cost parameter with either linear or radial kernel) and *K* (neighbourhood size in KNN) were: *λ*=0.0304, *C*_*SVML*_=0.0131, *C*_*SVMR*_=0.9401 and *K*=6.18 in the Brown Swiss population, and *λ*=0.00091, *C*_*SVML*_=0.0164, *C*_*SVMR*_=0.7352 and *K*=5.75 in the Fleckvieh population.

The average number of SNPs selected in the predictive model by the Lasso penalization was 7.5 and 25.9 in Brown Swiss and Fleckvieh respectively (min 3 and 14, max 19 and 51 in the two breeds).

The computation time was quite different in relation to the population size and classification method. The total time taken to complete one replicate of the classification procedure was as short as 3.51 seconds with Lasso logistic regression in Brown Swiss, and as long as 1 655.55 seconds (27.6 minutes) with KNN in Fleckvieh. In both breeds, Lasso logistic regression was the fastest method, while MAG and KNN were the slowest methods in Brown Swiss and Fleckvieh, respectively. SVM took intermediate times to compute, with the linear kernel being more than two-fold faster than the radial kernel. The computation time grew approximately linearly with sample size (Brown Swiss vs Fleckvieh) for all methods, except for KNN, for which it grew approximately quadratically.

Table 3 gives a complete overview of the time needed to run the five classification models in the two breeds.

**Table 3:** Time (in seconds) needed to complete 1 run of prediction with the five predictive models (Lasso, KNN, SVML, SVMR, MAG) in Brown Swiss and Fleckvieh cattle

Method	Brown Swiss	Fleckvieh
Lasso	3.51	25.01
KNN	38.71	1 655.55
SVML	10.16	115.89
SVMR	26.01	248.24
MAG	59.31	264.08

## Discussion

In this paper, a general framework for the identification of mutation carriers from SNP genotypes has been described. In a previous publication [21], SNP genotypes were used to predict carriers of the BH2 haplotype in cattle populations. The BH2 haplotype has been associated to stillbirth and peri-natal mortility in calves. The mutation behind BH2 has in the meantime been characterized (point substitution in the *TUBD1* gene, see [23] for details), and the step forward towards the prediction of carriers of the actual mutation — rather than of the associated haplotype — is presented in this paper.

The use of SNP genotypes to identify mutation carriers builds on special characteristics of the genome around the mutation, i.e. SNP loci in linkage disequilibrium and in recombination phase with the mutation. This approach has proved to be very accurate: in this study, the accuracy of identifying mutation carriers was higher than 95 *%* in both cattle breeds. These results are in line with those from similar studies (e.g. test error rate ≤1 *%* for BH2 haplotype in cattle [21], 0−5 *%* for HLA alleles in humans [24], ∼6 *%* for casein alleles in cattle [19]), and confirm that this is a highly effective approach with accuracy potentially close to 100 *%* (virtually faultless) for practical applications in animal genetics.

Lasso-penalized logistic regression, support vector machines with linear kernel (SVML) and multi-allelic gene prediction (MAG) gave the highest prediction accuracy both in Brown Swiss and Fleckvieh cattle, with limited variation over replicates. KNN and SVMR, on the other hand, showed a substantially lower and more variable prediction accuracy. The fact that more flexible models like KNN and SVMR perform worse than Lasso-penalized logistic regression, SVML and MAG indicates that the decision boundary in this classification problem is probably linear. KNN is a non-parametric method that accomodates to the local structure of the data. The use of a radial kernel in support vector machines tends to give small weight to observations which are far — in terms of Euclidean distance — from the classification candidate and, conversely, large weight to closer observations (thereby displaying a “local” behaviour). KNN and SVMR are therefore designed for non-linear classification problems, when the decision boundary between classes is far from linear and they’re expected to outperform linear methods under these circumstances. The linearity of the decision boundary therefore, accounts — at least partly — for the relatively worse performance of KNN and SVMR.

Among the tested classification methods, MAG is the only one that makes use of reconstructed haplotypes around the mutation. The additional information from phased SNP genotypes proved to be helpful especially in classifying observations belonging to the least frequent class (non-carriers in Brown Swiss, carriers in Fleckvieh). Despite a slighlty lower TPA (≲1 *%*), MAG showed TNR $\gtrsim 1.6\, \% \-- 0.8\, \%$ in Brown Swiss and TPR $\gtrsim 3.5\, \% \-- 7.2 \,\%$ in Fleckvieh compared to Lasso and SVML. Reconstructing haplotypes, however, though relatively inexpensive under some circumstances, adds complexity to the problem. Lasso and SVML may offer a valid alternative, which is particularly attractive from the machine/statistical learning perspective where a matrix of “features” (variables) is used to predict “labels” (observations).

In terms of computation time, Lasso logistic regression was by far the fastest method, followed by SVML and SVMR. MAG and KNN were the slowest methods with the Brown Swiss and Fleckvieh datasets, respectively. Additionally, KNN computation time appears to grow quadratically with problem size, while all other methods behaved linearly in terms of computation time as function of data size. In this work, no efforts were made to optimise the implementation of the different algorithms and computation strategies, and therefore the reported computation times are only indicative. Still, they can provide valuable information and useful guidelines as to the relative expected performance of the different classification methods.

### Fleckvieh vs Braunvieh

The identification of mutation carriers proved to be very accurate in both breeds. Looking at results from Lasso, SVML and MAG – the best performing classifiers — the total prediction accuracy (TPA) was 0.998, 0.996 and 0.994 in Fleckvieh, and 0.988, 0.991 and 0.983 in Brown Swiss. Carriers of the mutation (TPR) were identified with accuracy 0.956, 0.924 and0.991 in Fleckvieh, and 1.000, 0.998 and 0.992 in Brown Swiss. The accuracy to identify non-carriers of the mutation (TNR) was 0.999, 0.999 and 0.998 in Fleckvieh, and 0.968, 0.976 and 0.984 in Brown Swiss. The variability of estimated accuracy was very limited, lower than 5 *pp* in all cases. Overall, TPA and TNR were slightly higher in Fleckvieh, while TPR was substantially higher in Brown Swiss: i.e. it was relatively more difficult to identify mutation carriers in the Fleckvieh dataset, non-carriers in the Brown Swiss dataset. In particular, the identification of carriers showed highly variable accuracy in the Fleckvieh (TPR ≂0.99 with MAG, ≲0.95 with Lasso and SVML, and as low as 0.775 and 0.288 with SVMR and KNN), whereas in Brown Swiss also non-carriers were quite accurately identified (TNR >0.96 with Lasso, SVML, SVMR and MAG; TNR ≈0.8 with KNN).

The different results observed in the two breeds, can have both a statistical and a population genetics interpretation. First, there is sample size. As a general rule, prediction accuracy will asymptotically reach 100 % with increasing sample size [35]: more data would increasingly cause fewer overfitting problems and reduce the need for penalization. In the field of genomic predictions, the size of the reference population is known to influence the accuracy of GEBVs (genomic estimated breeding values) [36]. In the case of prediction of mutation carriers, this is nicely illustrated by Pirola et al. [19] who showed how the prediction error and variance are inversely related to sample size. In the present study, the sample size was about ten times higher in the Fleckvieh (3116 animals) compared to the Brown Swiss (392 animals) breed. Another statistical aspect of importance is the class ratio. Unbalanced data are a known issue in binary classification problems [37]: when the two classes are not equally represented it is much harder to predict observations belonging to the minority class (the least represented class), and an overall high prediction accuracy could mask very poor performance in the minority class. The class ratio was very unbalanced in the Fleckvieh (1:25), much less so in the Brown Swiss (1:1.8).

**Fig. 4:**
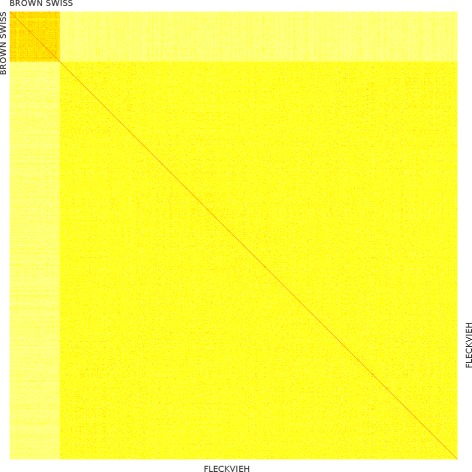
Heatmap of genomic relationships within and between breeds. Genetic relatedness estimated from SNP genotypes in Brown Swiss (*top-left*) and Fleckvieh (*bottom-right*) cattle. Darker colors correspond to higher estimated genomic relationships

**Fig. 5:**
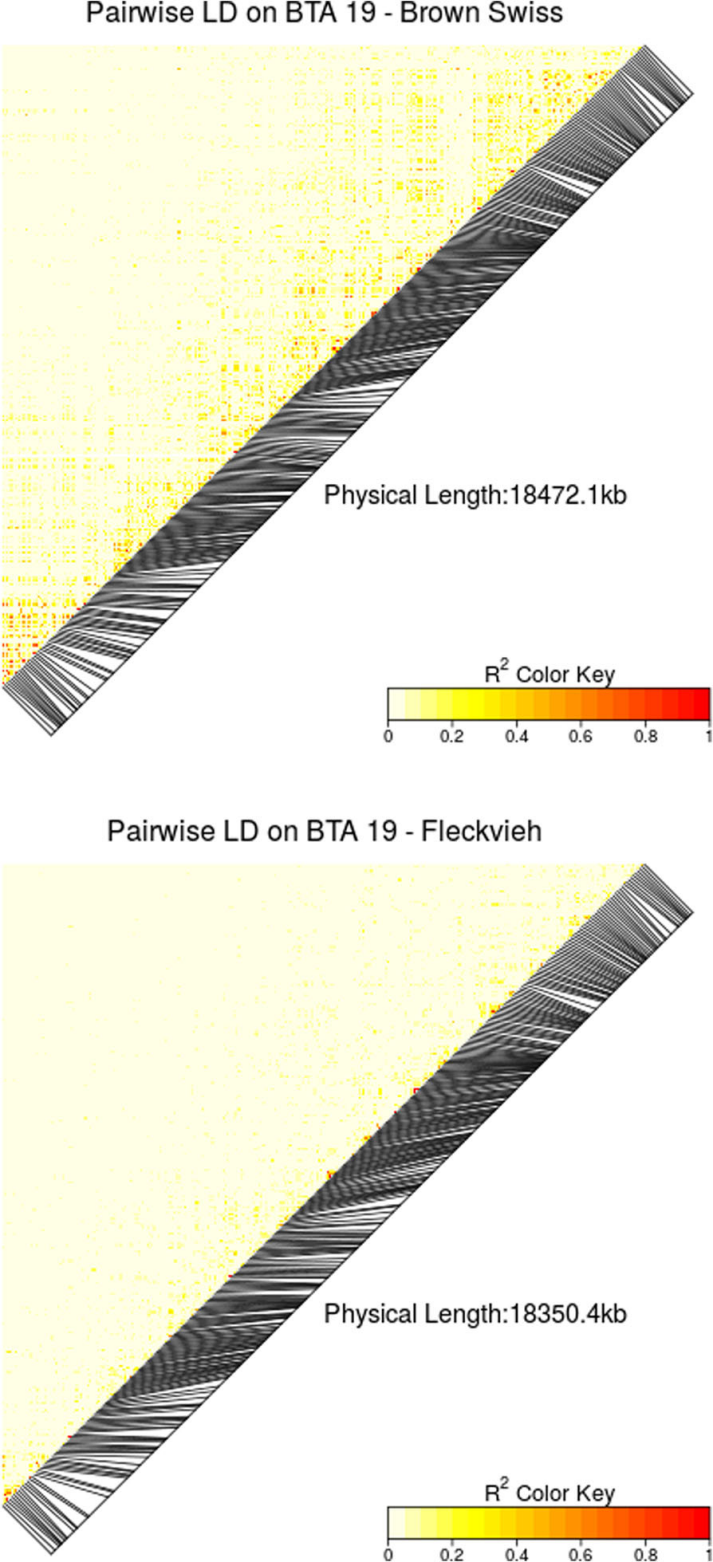
Linkage disequilibrium around the mutation on BTA19. *r* ^2^ values in the region 1 - 20 Mbps on BTA19 in Brown Swiss (*top*) and Fleckvieh (*bottom*) cattle. The *TUBD1* mutation is at 11 063 520 bps

More specifically linked to the genetic characteristics of populations are two additional aspects. On one hand, there are the genomic relationships among individuals; the accuracy of genomic predictions have long been found to be a function of genetic relatedness: “ceteris paribus”, stronger genetic links between the training and validation animals lead to higher accuracy of predictions (e.g. [38]). From SNP genotypes, higher average genomic relationships (à la Van Raden [39], rescaled to be in the range [ 0−2]) were estimated in Brown swiss (0.421±0.119) rather than in Fleckvieh (0.339±0.101); the heatmap in Fig. 4 shows the genomic relationships estimated within and across the two breeds. It can be argued that for predictions on a single locus “local” instead of genome-wide relationships are more meaningful. Average genomic relationships were therefore re-estimated using only SNPs around the *TUBD1* mutation (the first 20 Mbs on BTA19). Local relationships were higher than genome-wide relationships in both breeds: still, Brown Swiss showed higher local relatedness than Fleckvieh (0.4981 vs 0.4082). Another genetic aspect, the linkage disequilibrium (LD) between SNP markers and relevant loci/QTLs is known to influence the accuracy of genomic predictions: a sufficiently high LD is required for SNP-based predictions to be reliable [36]. The LD around the *TUBD1* mutation (first 20 Mbps on BTA19) was estimated as *r* ^2^ [40]: the average *r* ^2^ was 0.051 (interquartile range, IQR: [0.003−0.054]) in Brown Swiss and 0.022 (IQR: [ 0.001−0.026]) in Fleckvieh. Besides, better defined LD patterns and blocks around the mutation on BTA19 could be identified in Brown Swiss compared to Fleckvieh (Fig. 5).

Summarizing, sample size played in favour of the Fleckvieh dataset, while class ratio, genetic relatedness and linkage disequilibrium were more favourable in the Brown Swiss dataset. This helps explain the generally better predictive performances found in the Brown Swiss breed in this study.

### Error decomposition: variance and bias

The error incurred when making predictions can always be decomposed into its variance and bias [41]. The variance is the part of the error due to the variability of the predictor ($E[\hat {f}(x)-E(\hat {f}(x))]^{2}$); the bias refers to the systematic error, i.e. the expected value of the difference between predicted and true values ($E[\hat {f}(x)-f(x)]$).

For binary classification problems, with the misclassification loss function, the variance and bias of the classification error can be estimated as follows [42]:

9 $$\begin{array}{@{}rcl@{}} {}Var[\hat{p}(x)]=|2 \cdot \hat{p}(x)-1| I\left[(\hat{p}(x) \geq 0.5\, \&\, \bar{p}(x) < 0.5)\right. \\ \left. || (\hat{p}(x)<0.5 \; \& \; \bar{p}(x) \geq 0.5)\right] \end{array} $$

10 $$\begin{array}{*{20}l} {}Bias[\hat{f}(x)]=|2 \cdot p(x)-1| \, I[(p(x) \geq 0.5\, \& \, \bar{p}(x) < 0.5)  \\ || \; (p(x)<0.5\, \&\, \bar{p}(x) \geq 0.5)] \end{array} $$

We estimated the variance and bias of the classification error given by the five models (LASSO, SVML, SVMR, KNN, MAG) used to identify mutation carriers in the two breeds. Results are in Table 4. The variance was in the range between 0.00007 (FPR, Lasso) and 0.04131 (FNR, SVML) in Fleckvieh, and between 0.0001 (FNR, MAG) and 0.03306 (TER, KNN) in Brown Swiss. The bias ranged from 0.00015 (FPR, Lasso) to 0.72386 (FNR, KNN) in Fleckvieh, and from 0.00021 (FNR, SVML) to 0.21079 (FPR, KNN) in Brown Swiss. Overall, most of the error was taken up by the bias component. This says that the developed classifers make very stable predictions, even when resampling the training and validation sets (which changes the coefficients of the model). This is a desirable property, especially when the error rate is as low as that reported in this study: a small bias is not bound to severely affect the accuracy of prediction. A bias larger than the variance of the error partly stems out of class imbalance. With unbalanced data, most errors occur in the minority class, which means they are systematically misclassified. For instance, for KNN predictions in Fleckvieh, the errors in the minority class (FNR, Fig. 3) are in a well delimited area around the target, with little variation.

**Table 4:** Variance and bias of prediction error

	Method	TER			FNR			FPR		
		Error	Var	Bias	Error	Var	Bias	Error	Var	Bias
Brown	KNN	16.3 *%*	0.03306	0.13923	13.8 *%*	0.02989	0.09918	20.6 *%*	0.02136	0.21079
	Lasso	1.02 *%*	0.00031	0.00962	0.00 *%*	-	-	3.02 *%*	0.00087	0.02653
	SVML	0.91 *%*	0.00161	0.00873	0.12 *%*	0.00101	0.00021	2.41 *%*	0.00267	0.02367
	SVMR	2.15 *%*	0.00221	0.20115	1.11 *%*	0.00258	0.02264	3.92 *%*	0.00153	0.15731
	MAG	1.71 *%*	0.00423	0.01513	0.81 *%*	0.00010	0.01180	1.62 *%*	0.01178	0.02107
Fleckvieh	KNN	2.96 *%*	0.00143	0.02981	71.8 *%*	0.02688	0.72386	0.16 *%*	0.00021	0.00167
	Lasso	0.22 *%*	0.00011	0.00192	4.48 *%*	0.00115	0.04245	0.04 *%*	0.00007	0.00015
	SVML	0.37 *%*	0.00043	0.00251	7.63 *%*	0.04131	0.04083	0.07 *%*	0.00014	0.00093
	SVMR	0.93 *%*	0.00046	0.00571	22.5 *%*	0.01024	0.09911	0.05 *%*	0.00011	0.00186
	MAG	0.64 *%*	0.00094	0.00487	0.98 *%*	0.00101	0.06040	0.24 *%*	0.00097	0.00252

*KNN* k-nearest neighbors, *Lasso* *ℓ*1-penalized logistic regression, *SVML* support vector machine with linear kernel, *SVMR* support vector machine with radial kernel, *MAG* multi-allelic gene prediction

As a proportion of the error, the bias was larger in Brown Swiss than in Fleckvieh, for all error types (2.19, 4.24 and 1.95 times larger for TER, FPR and FNR, respectively). In Brown Swiss, the number of parameters (*p*) in the model (SNP) exceeded the number of observations (*n*) in the training set (*p*>*n*), unlike in Fleckvieh (*n*>*p*): this calls for stricter regularization in the Brown Swiss data, which would tip the bias-variance trade-off more towards the bias [41].

### Effect of sample size on prediction accuracy

The size of the reference population (individuals with both genotypic and phenotypic information) is known to play a major role in determining the accuracy of genomic predictions (e.g. [36]); this was shown also for the problem of predicting the carrier of specific gene alleles [19]. To investigate this in the present work, both available cattle populations (Fleckvieh and Brown Swiss) were reduced by randomly sampling 75 %, 50 % and 25 % of the animals. On the reduced subsets, Lasso, SVML and MAG were applied to identify carriers of the TUBD1 mutation, applying the same procedure as in Fig. 1 (except that for Brown Swiss 5-fold instead of 10-fold cross-validation was used to tune hyperparameters), repeated 10 times.

Results are reported in Table 5: TPA decreased by 11.2 % (SVML), 4.3 % (Lasso) and 0.5 % (MAG) in Brown Swiss and by 2.2 % (SVML), 1.8 % (Lasso) and 0.1 % (MAG) in Fleckvieh, when going from the full dataset to 25 % of the data. The larger drop in accuracy in Brown Swiss is likely due to the initial smaller sample size (392 vs 3116 animals). Also the standard deviation of TPA increases whith decreasing sample size, which suggests that predictions become progressively less reliable. When looking at TPR and TNR, it is clear that with smaller sample sizes predictions in the minority class get dramatically worse (in Brown Swiss and Fleckvieh respectively: by 24.8 % and 59.4 % with SVML, by 7.8 % and 12.4 % with Lasso, and by 2.8 % and 9.6 % with MAG), whereas predictions in the majority class are practically unaffected. This indicates that when the training population gets small, the trained binary classifier starts losing predictive power and begins being dominated by the most frequent class. Overall, MAG appears to be the most resilient method to data reduction, among those tested.

**Table 5:** Prediction accuracy with Lasso, SVML and MAG when the sample size is reduced from 100 to 25 % of the original size

	Breed	Reduction	TPA	TNR	TPR	LD	K	locK
SVML	Brown Swiss	1.00	0.992 (0.0081)	0.981 (0.0237)	0.998 (0.0047)	0.0469	0.4212	0.4981
	Brown Swiss	0.75	0.984 (0.0440)	0.983 (0.0521)	0.986 (0.0516)	0.0584	0.4165	0.4987
	Brown Swiss	0.50	0.920 (0.1745)	0.871 (0.2961)	0.981 (0.0285)	0.0626	0.4237	0.4894
	Brown Swiss	0.25	0.880 (0.1562)	0.738 (0.3751)	0.977 (0.0653)	0.0690	0.4183	0.4992
	Fleckvieh	1.00	0.996 (0.0025)	0.999 (0.0009)	0.928 (0.0525)	0.0226	0.3392	0.4082
	Fleckvieh	0.75	0.995 (0.0034)	0.998 (0.0014)	0.909 (0.0857)	0.0228	0.3252	0.4081
	Fleckvieh	0.50	0.991 (0.0062)	1.000 (0.0001)	0.814 (0.1239)	0.0234	0.3195	0.3952
	Fleckvieh	0.25	0.974 (0.0256)	1.000 (0.0003)	0.377 (0.2126)	0.0241	0.3197	0.4078
Lasso	Brown Swiss	1.00	0.987 (0.0112)	0.966 (0.0292)	1.000 (0.0000)	0.0469	0.4212	0.4981
	Brown Swiss	0.75	0.986 (0.0338)	0.972 (0.0640)	1.000 (0.0000)	0.0596	0.4176	0.4776
	Brown Swiss	0.50	0.967 (0.0472)	0.913 (0.1405)	1.000 (0.0000)	0.0586	0.4078	0.4862
	Brown Swiss	0.25	0.945 (0.1117)	0.891 (0.1329)	0.958 (0.1443)	0.0683	0.4526	0.5151
	Fleckvieh	1.00	0.997 (0.0017)	1.000 (0.0000)	0.942 (0.0369)	0.0226	0.3392	0.4082
	Fleckvieh	0.75	0.996 (0.0034)	1.000 (0.0000)	0.944 (0.0869)	0.0229	0.3235	0.4050
	Fleckvieh	0.50	0.987 (0.0309)	0.988 (0.0329)	0.923 (0.1545)	0.0248	0.3000	0.3876
	Fleckvieh	0.25	0.979 (0.0247)	0.989 (0.0002)	0.825 (0.2020)	0.0250	0.3106	0.3961
MAG	Brown Swiss	1.00	0.984 (0.0152)	0.982 (0.0163)	0.992 (0.0112)	0.0469	0.4212	0.4981
	Brown Swiss	0.75	0.983 (0.0155)	0.976 (0.0327)	0.989 (0.0161)	0.0589	0.4178	0.4832
	Brown Swiss	0.50	0.982 (0.0194)	0.973 (0.0393)	0.987 (0.0259)	0.0586	0.4222	0.4891
	Brown Swiss	0.25	0.978 (0.0352)	0.955 (0.0776)	0.992 (0.0331)	0.0683	0.4446	0.5097
	Fleckvieh	1.00	0.994 (0.0030)	0.998 (0.0021)	0.991 (0.0030)	0.0226	0.3392	0.4082
	Fleckvieh	0.75	0.994 (0.0032)	0.998 (0.0024)	0.939 (0.0603)	0.0225	0.3271	0.4069
	Fleckvieh	0.50	0.994 (0.0048)	0.997 (0.0031)	0.917 (0.0871)	0.0239	0.3095	0.3962
	Fleckvieh	0.25	0.993 (0.0074)	0.997 (0.0042)	0.896 (0.1438)	0.0251	0.3102	0.3960

*LD* average linkage disequilibrium estimated around the mutation, *K* average genome-wide genomic relationships, *locK* average genomic relationships estimated using only SNPs around the mutation

The size of the training population is confirmed to be a major factor behind the accuracy of genomic predictions, also in the identification of carriers of recessive mutations.

At each size-reduction step, the LD (*r* ^2^) around TUBD1, and the genome-wide and “local” (around TUBD1) rescaled genomic relationships à la Van Raden were estimated. Around the mutation was again defined by taking SNPs from the first 20 Mbps on BTA19. The estimated LD around the mutation appears to increase with decreasing sample size; however, LD estimates are known to be biased upwards by small sample size in cattle [43]. Both genome-wide and local genomic relationship are little affected by sample size. This indicates that the drop in accuracy is explained mainly by the size of the training set and apparently not by a lower LD between SNP and the mutation, or by looser relationships between animals.

### Extension to another harmful recessive mutation

From an indipendent population of Italian Brown Swiss cattle (provided by the Italian Brown Association: www.anarb.it), three hundred and five Brown Swiss bulls typed for congenital spinal dysmyelination (SDM, [44]) were available to test the accuracy of identifying mutation carriers from SNP genotypes. SDM is another recessive mutation of interest in cattle, caused by a missense mutation in the SPAST gene on BTA 11 [45]. All bulls (8 carriers, 297 non-carriers) were genotyped with the BovineSNP50 v2 (54 K) Illumina BeadChip (2442 SNPs on BTA11, after editing).

The three classification methods that gave the best results on the TUBD1 mutation were applied to the identification of carriers of the SDM mutation: Lasso, SVML and MAG. As in Fig. 1, 80 *%* of the data were used to train the classifier, and the remaining 20 *%* to test it. Given the smaller dataset size (∼240 animals for training), a 60/40 cross-validation scheme was used to tune the hyperparameters (*λ*, *C*). The procedure was repeated 100 times to estimate prediction accuracy. With Lasso, SVML and MAG, respectively, TPA was 0.987 (±0.018), 0.978 (±0.016) and 0.983 (±0.016); TPR was 0.625 (±0.342), 0.435 (±0.356) and 0.611 (±0.341); and TNR was 0.997 (±0.007), 0.999 (±0.003) and 0.993 (±0.011).

Overall, the identification of carriers proved to be effective also when tested on a different mutation. However, compared to the results with the TUBD1 mutation, a higher test error rate was estimated for SDM, especially in the minority class (carriers of the mutation): whereas TPA and TNR were both very high and close to 100 *%*, TPR was quite lower, in the range 0.435−0.625. Probably, this is related to the different frequency of carriers: 4.04 *%* (Fleckvieh) and 63.78 *%* (Brown Swiss) for TUBD1, as low as 2.62 *%* for SDM. Highly unbalanced data are expected to yield worse predictive performances. This was especially true in the case of SDM for which there were only eight carriers. The smaller sample size too, is likely to have played a role in the higher prediction error rates and, especially, the larger variability of estimates, as we showed when reducing the size of the TUBD1 dataset (Table 5).

Unlike the TUBD1 mutation, MAG did not (slightly) outperform Lasso and SVML in the minority class: using haplotypes did not appear to be as beneficial with the SDM mutation. This may be related to the different degree of concordance between the mutation and the associated haplotype: this was 99.2 *%* between TUBD1 and the BH2 haplotype [23], and 94.4 *%* between SDM and the BHD haplotype [9].

### Prediction accuracy with the low density SNP chip

Several SNP-chips are available in cattle, with different marker densities and costs (see [46]). In this work, an overall very high accuracy of prediction was achieved using the BovineSNP50 v2 Illumina BeadChip, which contains 54 609 SNPs. It would be interesting to see what would be the accuracy of identifying mutation carriers using lower density SNP-chips. In particular, it is of interest to verify whether comparable accuracies could be achieved with fewer SNPs. Since genotyping animals is a cost, especially for commercial farms, low density SNP chips may offer a cheaper alternative for the identification of mutation carriers.

From the 54 K SNP-chip data, the SNP on BTA19 corresponding to the BovineLD v2 Illumina BeadChip (185 and 211 SNPs in Brown Swiss and Fleckvieh, respectively) were extracted and used for classification. Lasso and SVML, were used for classification, with the same experimental design as in Fig. 1 (10-fold cross-validation, 100 replicates).

With Lasso, TER, FNR and FPR were 0.0183 (±0.0151), 0.009 (±0.0065) and 0.0350 (±0.0287) in Brown Swiss, and 0.0111 (±0.0036), 0.0605 (±0.0309) and 0.0049 (±0.0037) in Fleckvieh. With SVML, TER, FNR and FPR were 0.0167 (±0.0146), 0.0109 (±0.0145) and 0.0257 (±0.0294) in Brown Swiss, and 0.0116 (±0.0042), 0.228 (±0.0959) and 0.0025 (±0.0023) in Fleckvieh. The computation time for one replicate of the model in the Brown Swiss and Fleckvieh datasets, respectively, was 3.51 and 5.58 s using Lasso, and 7.88 and 33.55 s using SVML.

Overall, performances are comparable with the 54 SNP-chip: only the identification of carriers in Fleckvieh was notably less accurate with lower densitiy SNP data.

### Localize mutation via resampling

In predictive models, it can be of interest to find out which variables are more relevant for accurate predictions. When resampling strategies are adopted, the relevance of individual predictors can be indirectly derived from the frequency with which they appear in the different replicates of the predictive model. In the case of SNP-based models, the most predictive SNPs are likely to be linked to the target QTL/mutation. This approach was already presented by Biffani et al. [21] to track the position of the BH2 haplotype. The BH2 haplotype is located at 10.140−−11.049 Mb on BTA19 [22], and could be accurately identified through the resampling approach. However, SNP data were used both to first reconstruct the BH2 haplotype and then localize it. Additionally, it may be harder to localize a point mutation rather than a longer associated haplotype. Therefore, a similar resampling approach was adopted here to map the position of the *TUBD1* mutation on BTA19.

**Fig. 6:**
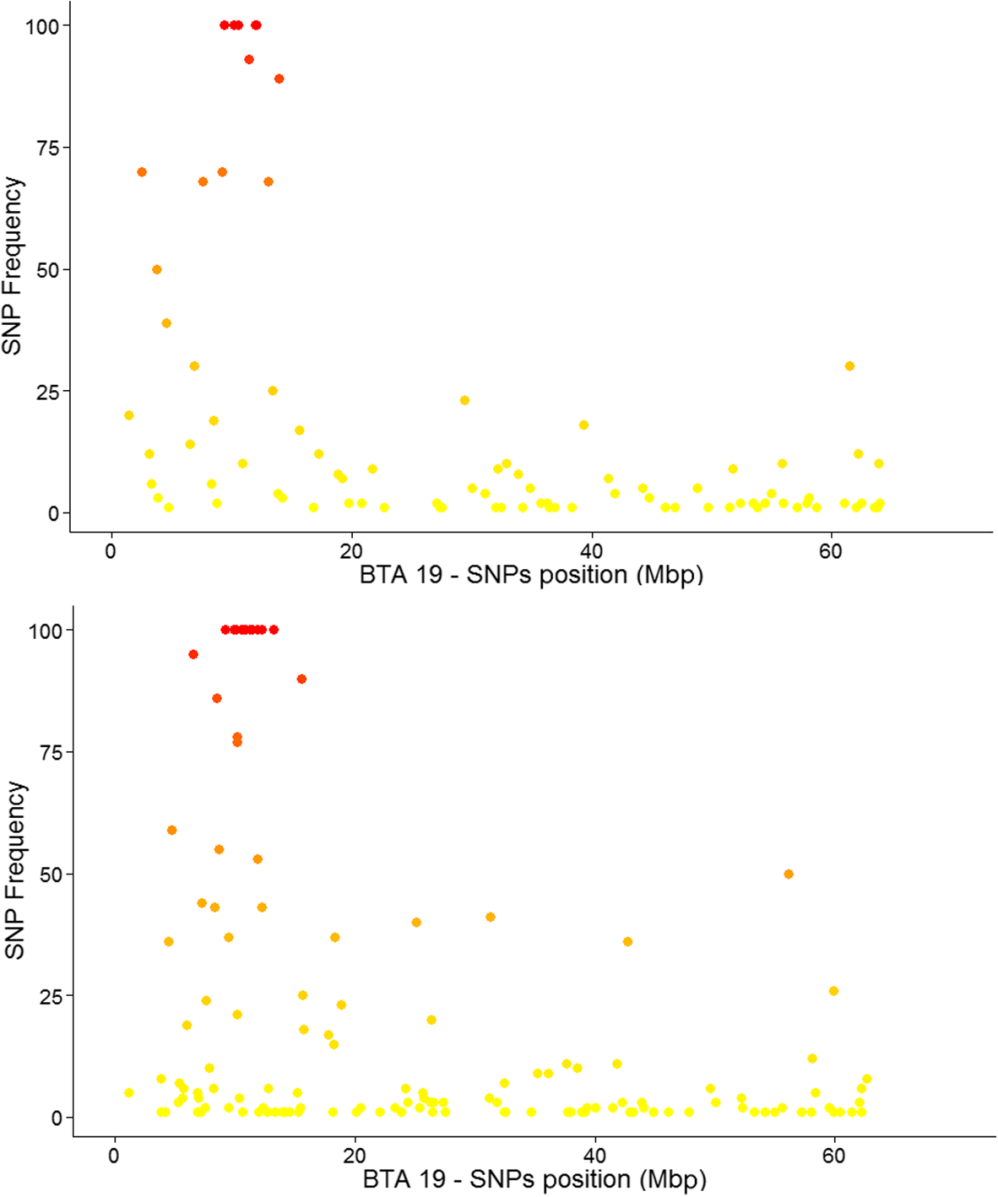
Predictive SNPs selected by Lasso-penalization from the 50K SNP-chip. Frequency of inclusion of SNPs in the Lasso-penalised logistic regression model for each of the 100 replicates, plotted against the SNP position on BTA19 (*Top*, Brown Swiss; *bottom*, Fleckvieh)

The lasso penalised logistic regression model in Eq. 1 has the property of selecting, through cross-validation, a subset of predictive SNPs. This subset can vary from replicate to replicate. In Fig. 6, the absolute frequencies of inclusion of SNPs in the predictive model were plotted against their bps positions along BTA19, separately in Brown Swiss (top) and Fleckvieh (bottom). The *TUBD1* mutation is located at 11 063 520 bps [23], which correspond to where the frequency peaks appear in both breeds (Fig. 6): the mutation could thus be localized by mining classification results, without any prior information on its position.

**Fig. 7:**
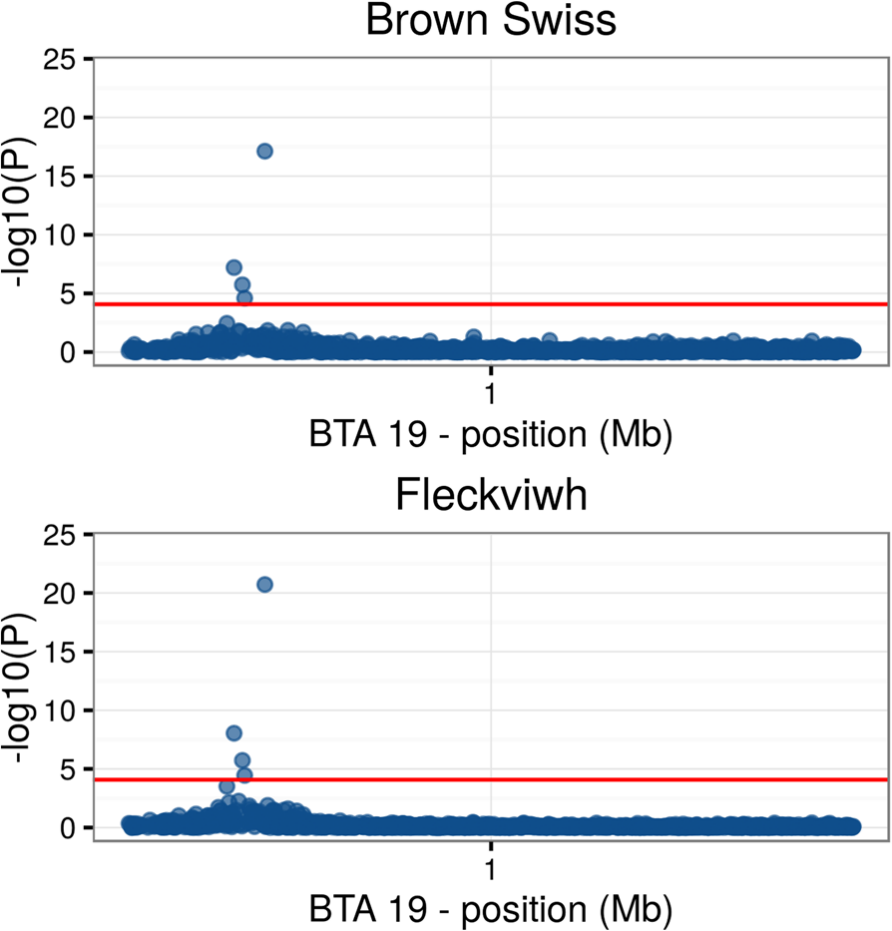
Manhattan plot of −*log*(*p*−*value*). Results from a GWAS analysis for carriers/non-carriers of the TUBD1 mutation. Brown Swiss above, Fleckvieh below

To provide a comparison, *p*-values from a genome-wide association study (GWAS) for the binary trait carrier/non-carrier of the TUBD1 have been plotted in Fig. 7. A logistic regression model for the probability of being carrier of the mutation was fitted for every single SNP on BTA 19, accounting for the polygenic effect through the matrix of genomic relationships. The GWAS approach also detected a strong signal of association at around 11 Mbps on BTA19. The significant associations had a lower *p*-value in Fleckvieh than in Brown Swiss, probably due to the much larger sample size in the former.

The results reported here and -previously- by Biffani et al. [21] show that resampling methods offer a valid alternative or complement to GWAS studies, as discussed by Biscarini et al. [47]. GWAS studies are in fact known to suffer from some limitations, like susceptibility to spurious associations and poor reproducibility of results [48, 49]. Combining the two approaches may lead to more robust association results.

### Applications to the management of cattle populations

In cattle genetics, causal mutations or associated haplotypes for inherited disorders are being discovered at unprecedented rates (see USDA list: [13]). It is therefore useful to have tools that allow breeders to identify carriers. The degree of association between haplotypes and underlying mutations is variable [9]: sometimes very high (e.g. JH1 haplotype and the *CWC15* gene in Jersey cattle), sometimes much looser (e.g. two versions of the haplotype associated with complex vertebral malformation in the Holstein breed, one with and one without the causative mutation). This may increase the uncertainty of identifying mutation carriers from SNP genotypes, and there is therefore value in directly predicting mutation rather than haplotype carriers.

In this paper, we showed that mutation carriers can be identified very accurately from SNP genotypes. Bulls and cows in breeding programmes are often routinely genotyped, e.g. for parentage verification, estimation of genomic breeding values, and it would therefore be rather straightforward and inexpensive to use already available genotype data to identify mutation carriers. Using the 54 K SNP-chip, the accuracy of prediction has been shown to be very high, close to 100 % in both breeds, with very few misclassifications also in the minority class. At lower SNP densities the overall accuracy was still impressively high, slightly lower than 99 *%*, but larger proportions of misclassifications were observed in the minority class (false positives in Brown Swiss, false negatives in Fleckvieh). In order to maximise prediction accuracy in all classes while keeping costs low, mixed genotyping strategies can be adopted by which most of the population is genotyped at low densities and only relatively few animals are genotyped with the 54 K chip: 54 K SNP genotypes can then be imputed back in all animals [50]. Mixed genotyping strategies are current standard practice in several national dairy selection schemes (e.g. Canada [51]). To assess the effect on prediction accuracy of imputing back to higher SNP densities, different proportions of extracted BovineLD SNP genotypes were imputed back to 54K SNP genotypes: 25, 50 and 75 %, and Lasso was run on each of them for the identification of carriers. Very similar results to those reported in Table 2 were obtained. For instance, with 75 % of the animals genotyped at low densities, TPA, TNR and TPR were still 0.986, 0.962 and 1.000, respectively. This may be related to the high imputation accuracy in dairy cattle [52].

With the size of the data used in the present work (∼3000 animals), Lasso and SVML were quite efficient with an *R* implementation. For larger datasets, though (e.g. hundreds of thousands of genotyped animals like the US Holstein population), scalability would certainly be an issue, and more efficient implementations of the algorithms, combined with computation strategies like parallel computing on multiple cores or distributed computing on a computer cloud/cluster should be adopted. A popular combination in machine learning and “big data” analysis is given by the scripting language Python within the Apache Spark framework for distributed computing [53].

Once mutation carriers have been identified, this information can be used in breeding strategies to guide selection decisions and plan matings, with the objective of reducing the frequency of carriers in the population and avoiding mating of carriers. Mate allocation schemes to control inbreeding and recessive disorders have been already proposed in cattle [54, 55]. Besides breeding, the accurate identification of mutation carriers can be useful also in conservation programmes for marginal breeds at risk of extinction. These breeds typically have higher inbreeding (e.g. Chillingham [56]), which exposes them to a higher incidence of recessive genetic diseases. Avoiding the mating of carriers would therefore be very beneficial.

## Conclusions

This paper shows that SNP genotypes can be used very effectively to predict carriers of harmful recessive mutations in cattle populations. The *TUBD1* mutation on BTA19, associated with reduced fertility in cows, was chosen as illustration. Together with MAG, Lasso-penalized logistic regression and Support Vector Machines with linear kernel gave the lowest error rates indicating the probable linear nature of this classification problem. Overall, the prediction accuracy was very high, close to 100 *%* in both breeds. Compared to single SNP genotypes, the use of haplotypes gave better accuracy in the minority class when the haplotype-mutation concordance was close to 100 % (BH2-TUBD1). The opposite was true for looser associations between haplotype and mutation (SDM). When using the low-density SNP-chip, the total error rate was still very low (∼1 *%*), but the proportion of misclassifications in the minority class tended to increase (relatively many false positives in Brown Swiss, false negatives in Fleckvieh). This can however be compensated by optimised genotyping strategies combined with genotype imputation techniques, which could potentially make this a very effective and efficient tool for the identification of carriers of any mutation or haplotype of interest (both positive or negative) in *Bos taurus*. There is potential to build effective applications to be used by breeders and farmers (e.g. the Zanardi pipeline [57]). The presented procedure could in principle be extended to any other diploid organism, for agriculture applications in farm animals, crops and trees, and for medical applications in humans.

## Abbreviations

FNR: False negative rate
FPR: False positive rate
GEBV: Genomic estimated breeding value
IQR: Interquartile range
KNN: k-nearest neighbours
TER: Total error rate
SNP: Single nucleotide polymorphism
SVM: Support vector machines
SVML: SVM with linear kernel function
SVMR: SVM with radial kernel function
